# Divergent Modulation of Neuronal Differentiation by Caspase-2 and -9

**DOI:** 10.1371/journal.pone.0036002

**Published:** 2012-05-18

**Authors:** Giuseppa Pistritto, Veruska Papaleo, Pilar Sanchez, Claudia Ceci, Maria Luisa Barbaccia

**Affiliations:** 1 Department of Neuroscience, University of Rome Tor Vergata-Medical School, Rome, Italy; 2 Department of Biochemistry and Molecular Biology, Faculty of Medicine, University of Granada, Granada, Spain; University of South Florida, United States of America

## Abstract

Human Ntera2/cl.D1 (NT2) cells treated with retinoic acid (RA) differentiate towards a well characterized neuronal phenotype sharing many features with human fetal neurons. In view of the emerging role of caspases in murine stem cell/neural precursor differentiation, caspases activity was evaluated during RA differentiation. Caspase-2, -3 and -9 activity was transiently and selectively increased in differentiating and non-apoptotic NT2-cells. SiRNA-mediated selective silencing of either caspase-2 (si-Casp2) or -9 (si-Casp9) was implemented in order to dissect the role of distinct caspases. The RA-induced expression of neuronal markers, i.e. neural cell adhesion molecule (NCAM), microtubule associated protein-2 (MAP2) and tyrosine hydroxylase (TH) mRNAs and proteins, was decreased in si-Casp9, but markedly increased in si-Casp2 cells. During RA-induced NT2 differentiation, the class III histone deacetylase Sirt1, a putative caspase substrate implicated in the regulation of the proneural bHLH MASH1 gene expression, was cleaved to a ∼100 kDa fragment. Sirt1 cleavage was markedly reduced in si-Casp9 cells, even though caspase-3 was normally activated, but was not affected (still cleaved) in si-Casp2 cells, despite a marked reduction of caspase-3 activity. The expression of MASH1 mRNA was higher and occurred earlier in si-Casp2 cells, while was reduced at early time points during differentiation in si-Casp9 cells. Thus, caspase-2 and -9 may perform opposite functions during RA-induced NT2 neuronal differentiation. While caspase-9 activation is relevant for proper neuronal differentiation, likely through the fine tuning of Sirt1 function, caspase-2 activation appears to hinder the RA-induced neuronal differentiation of NT2 cells.

## Introduction

The human teratocarcinoma cell line Ntera2/cl.D1 (NT2 cells) represents a well-established model to study the retinoic acid (RA)-induced terminal differentiation of human neural progenitors into post-mitotic neurons (NT2-N) [1]–[3]. The many features that NT2-N share with human fetal neurons has generated great interest for their potential use as graft source for cell therapy in neurodegenerative diseases [4], a perspective that warrants a deep understanding of the molecular mechanisms underlying NT2 cell differentiation.

Caspases, **c**ysteine-dependent **aspa**rtate-specific protea**ses**, are classified according to phylogenetic relationships, structure, substrate specificity, location in signaling pathways (“initiator”, i.e. upstream activator of the apoptotic cascade, or “executioner”, i.e. effector of apoptosis) and function. The functional definition of “apoptotic” and “pro-inflammatory” caspases defines the two best-studied processes in which these proteases are operative, though it may not include all their possible functions [5], [6].

Apoptosis occurs massively in the developing brain, where it eliminates neurons that fail to reach their proper targets and helps shaping/refining neuronal networks. However, caspase’s implication in neurodevelopment may exceed the morphogenetic and “systems matching”–i.e. modulation of optimal connectivity between neurons and their targets or afferents– role fulfilled by apoptosis in the developing brain [7]. Indeed, following the seminal observation by Ishizaki et al. [8], the implication of caspases in the differentiation of diverse cell types, and particularly neurons, as well as in various aspects of neuronal plasticity, is becoming more accepted [9]–[11]. Across species, both “initiator” and “executioner” caspases appear involved in neuronal differentiation/maturation, and the evidence gathered thus far in the mammalian brain appears to suggest the ultimate involvement of caspase-3 [11]–[16]. Whether the latter is a necessary requirement or an epiphenomenon consequent to the hierarchical activation of caspases, as shown to occur following appropriate stimuli leading to apoptosis [5], is so far unclear.

Sirt1 is a NAD^+^-dependent class III histone/lysine deacetylase whose activity is implicated in chromatin remodeling, transcriptional silencing, stress response and cellular differentiation [17], [18]. Sirt1 also appears to regulate in a redox-dependent manner murine neural precursor differentiation, where conditions determining its activation or inhibition direct neural precursors towards the glial or the neuronal lineage, respectively, by controlling the expression of the proneural bHLH factor MASH1 [19]. Of particular relevance in this context, is the finding that, under apoptotic conditions, Sirt1 was shown to be cleaved by caspases-1, -3,-6, -8 and -9 [20].

Neuronal differentiation is relevant not only to shape the brain connectivity during development but also in the context of neurodegenerative diseases, where differentiation of resident neuronal progenitors may represent an adaptive approach to replace, at least in part, the neurons that are killed, though not exclusively, by caspase activation [7], [10]. Hence, as the available evidence suggests [11]–[16], caspases may behave as double edge swords in the pathophysiology of neurodegenerative diseases. Following this line of thinking, caspase’s pharmacological inhibition, albeit beneficial in reducing/slowing down neuronal death [21]–[24], theoretically may hinder the intrinsic brain neurogenic potential. Altogether, these considerations prompted us to evaluate whether and which caspases are operative in the differentiation of NT2 cells.

The present results show that although the activity of caspase-2, -3 and -9 is transiently increased during the RA-induced differentiation of NT2 cells, caspase-2 and 9 appear to play the most relevant, though opposite, roles in the process. In fact, siRNA-mediated silencing of caspase-9 delays/reduces the expression of neuronal markers in differentiating non-apoptotic NT2 cells. The silencing of caspase-9 expression also greatly reduces the cleavage of Sirt1 that occurs during NT2-cell differentiation. Hence, caspase-9 activation seems relevant for the proper RA-induced neuronal differentiation of NT2 cells, likely through the fine-tuning of Sirt1 function. In contrast, silencing of caspase-2 increases the expression of neuronal differentiation markers, suggesting that caspase-2 activation operates as a restraining mechanism on NT2 cell differentiation.

## Materials and Methods

### Cell Culture

NT2 cells (ATCC, Manassas, VA, USA) were grown in DMEM (Invitrogen, Carlsbad, CA, USA) supplemented with 10% fetal bovine serum (FBS), 1% glutamine and penicillin/streptomycin (10,000 UI/ml and 10 mg/ml, respectively) and were induced to differentiate following a cell aggregation protocol [25], with minor modifications [3]. Retinoic acid (1 µM) was added to the cultures twice weekly for 4 weeks. Post-mitotic neurons were obtained by replating the cells on Matrigel (BD, San Jose, CA, USA)-coated dishes, in fresh medium containing mitotic inhibitors (1 µM cytosine arabinoside, 10 µM fluorodeoxyuridine, 10 µM uridine) to reduce proliferation of non-neuronal cells. After 10–15 days of treatment with mitotic inhibitors the cultures contained >99% pure post-mitotic neurons (NT2-N).

### Removal of Apoptotic Cells

Non-apoptotic cells were purified from apoptotic ones by negative selection using the Apoptotic cell isolation kit (Biovision, CA, USA). Briefly, the cells were collected, washed twice with PBS cells and re-suspended in binding buffer containing Annexin V-biotin, according to the manufacturer’s instructions. After incubation at room temperature for 15 min in the dark, the cells were washed, re-suspended in binding buffer containing streptavidin-coated magnet beads and incubated for 15 min at room temperature. The apoptotic Annexin V-positive cells were separated from living cells using a magnetic surface (Dynal MCP-S, Invitrogen).

### Caspase Activity Assay

Quantitative enzymatic activity assays were carried out as previously described [26]. Fifty µg of total cell lysates were assayed for caspase activity using 7-amino-4-trifluoromethylcumarin (AFC)-conjugated peptide substrates: YVAD-AFC, VDVAD-AFC, DEVD-AFC, VEID-AFC, IETD-AFC and LEHD-AFC (Biovision, CA, USA) as specific for caspase-1, -2, -3, -5, -6, -8 and -9, respectively. Briefly, cytosolic cell lysates were incubated with each fluorogenic substrate in appropriate buffer for 2 h at 37°C. The peptide-AFC was hydrolyzed by the enzyme and the fluorescence measured using a Victor plate fluorometer (PerkinElmer, MA, USA) at Ex400/Em505 nm. Some assays were performed in the presence of broad spectrum caspase inhibitor Z-VAD-FMK (20 uM) (Biovision).

### siRNA Constructs and Stable Transfection of NT2 Cells

Kits of Ready-cloned HuSH 29mer shRNA against human caspase-2 and -9 in pGFP-V-RS plasmids were purchased from Origene (Rockville, MA, USA). Each kit included four shRNA constructs targeting different regions of each gene. NT2 cells were separately transfected with each construct to identify the shRNA that caused maximum inhibition for subsequent experiments. Cells were transfected using Lipofectamine 2000 (Invitrogen), following the manufacturer’s instructions. Forty-eight hours after transfection, cells were selected by adding 1 µg/ml puromycin. Stably transfected NT2 cells were maintained in medium containing 1 µg/ml puromycin. Silencing of caspase-2 or -9 gene was confirmed by RT-PCR and Western blotting for each clone that carried a stable transfection. The following shRNA sequences were selected and used for experiments in which caspase-9 and-2 were interfered, respectively: AGG ATT TGG TGA TGT CGA GCA GAA AGACC and CAA GGC CAC CTG GAG GAT ATG TTG CTC AC. Control NT2 cells also received a plasmid containing a non-effective 29-mer scrambled shRNA in pGFP-V-RS to take into account non-specific effects of the shRNA.

### Real Time PCR

NT2 cells were harvested at the indicated time points. Taqman quantitative real-time PCRs (NCAM, MAP2, TH, Mash1, GAPDH) were performed using commercially available Assay-On-Demand kits, n. Hs00941821_m1, Hs00258900_m1, Hs00165941_m1, Hs00269932_m1 and 4333764F, respectively (Applied Biosystems, Inc., Foster City, CA, USA). Total RNA was isolated from NT2 using the RNeasy plus mini-kit (Qiagen, Hilden, Germany) and the concentration was determined by spectrophotometry. For the generation of the first strand cDNA, 1 µg of total RNA was reverse transcribed by random primer extension using SuperScript III reverse transcriptase (Invitrogen) according to the manufacturer’s instructions. Gene expression assays were performed according to manufacturer’s instructions, with an ABI 7300 cycler. Triplicate C_T_ values were analyzed in Microsoft Excel using the comparative C_T_(ΔΔC_T_) method, according to the manufacturer’s instructions. The amount of target transcript (2^−ΔΔCT^) was obtained by normalizing the values to endogenous GAPDH.

### Antibodies and Western Blot Analysis

Antibodies: polyclonal anti-nCAM (R&D Systems, Minneapolis, USA), polyclonal anti-TH (Millipore, Billerica, MA, USA), polyclonal anti-caspase-9, monoclonal anti-caspase-2 and polyclonal anti-PARP (Cell Signal Technology, Danvers, USA), polyclonal anti-Sirt1 and monoclonal anti-βactin (Sigma, St Louis, USA), Goat anti-mouse and goat anti-rabbit HRP (GE Healthcare, Bio-Sciences Corp., NJ, USA) were used as secondary antibodies. Western blotting: NT2 cells were collected, lysed in RIPA buffer containing 25 mM Tris-HCl pH 7.6, 150 mM NaCl, 1% NP-40, 1% sodium deoxycholate, 0.1% SDS and protease inhibitors (protease inhibitor cocktail, Sigma). Protein concentration for each sample was determined by Bio-Rad protein assay reagent (Bio-Rad Laboratories, Inc, Richmond, CA, USA), separated by SDS-PAGE and transferred to PVDF blotting membrane. Membranes were washed in TBS-T buffer, blocked for 2 hr at RT with 5% fat free dried milk and incubated overnight with primary antibodies. After washing, the membranes were incubated with HRP-conjugated secondary antibody for 1 h at RT. The protein signals were visualized using the ECL-Plus Chemiluminescence kit (GE Healthcare, USA). Densitometric analysis was performed with TINA image software (version 2.09f, Raytest, Straubenhardt, Germany).

### Statistical Analysis

The data, representing the means ± SD of three experiments, each run either in duplicate or triplicate, were analyzed by ANOVA followed by post-hoc Dunnett test or by Kruskal-Wallis test, followed by post-hoc Student-Neuman-Keuls test, as appropriate.

## Results

### Caspase-2, -3 and -9 Activities Selectively Increase During RA-induced Neuronal Differentiation of NT2 Cells

**Figure 1:**
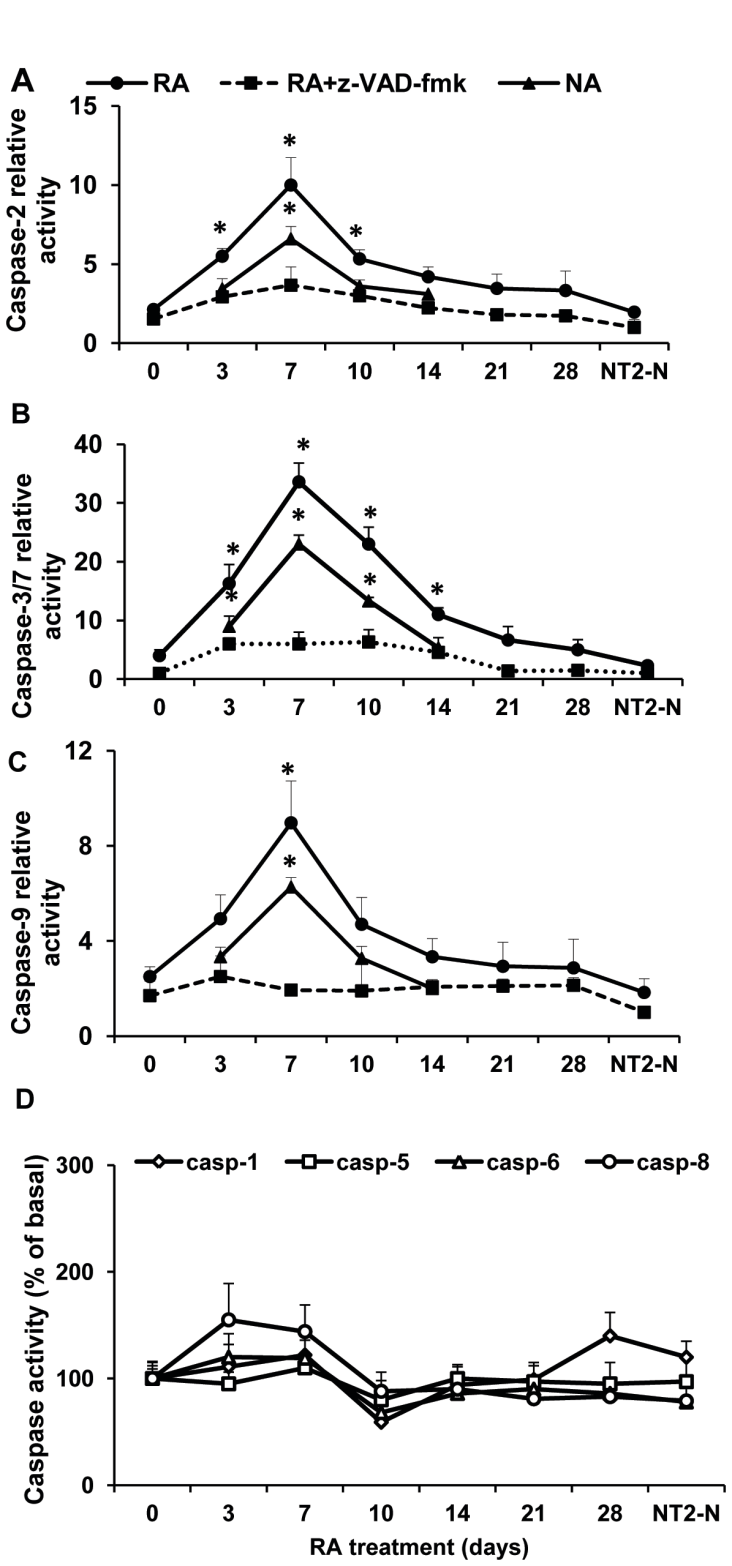
Caspase-2, -3 and -9 are selectively activated during RA-induced NT2 cell neuronal differentiation. Total population (RA, filled circles) and non-apoptotic (NA, filled triangles) were harvested at the indicated time points during RA treatment and their lysates were incubated for the assay of caspase-2 (A), -3 (B) and -9 (C) activity with the respective fluorogenic substrate (VDVAD-AFC, for caspase-2, DEVD-AFC, for caspase-3/7 and LEHD-AFC for caspase-9). Control samples, in the presence of the pan-caspase inhibitor Z-VAD-fmk, were run in parallel (Z-VAD-fmk, filled squares). Each point represents the mean ± SEM of 3 experiments, each run in duplicate. *P<0.05, compared to the respective value in undifferentiated NT2 cells (day 0 ). The activity of caspase-1, -5, -6 and -8, expressed as percentage of the respective basal activity in undifferentiated cells, failed to show significant changes throughout the RA-induced differentiation (D).

**Figure 2:**
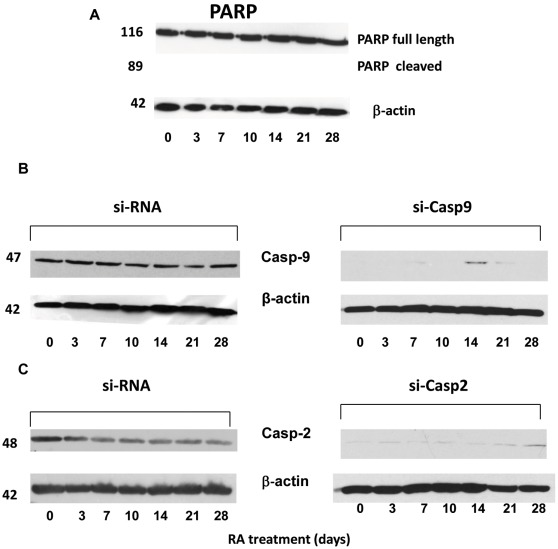
Lack of apoptotic activity in differentiating NT2 cells (A) and expression of caspase-9 (B) and caspase-2 (C) after si-RNA transfection of NT2 cells. Notwithstanding the increased caspase activity, no noticeable cleavage of poly-(ADP-ribose) polymerase (PARP) to its 89 kDa fragment was detected at any time point during NT2 cell differentiation (A). Specific si-RNA treatment ( see Material and Methods section) achieved an almost complete depletion of caspase-9 (B, si-Casp9) and caspase-2 (C, si-Casp2), that in both cases persisted throughout the RA-induced differentiation period of NT2 cells.

Among various caspases examined, only caspase-2, -3 and -9 were transiently but significantly activated during RA treatment (RA, Figure 1 A, B and C). For each caspase assay, control samples were run in parallel in the presence of the pan-caspase inhibitor z-VAD-fmk (z-VAD-fmk, Figure 1 A-C). As the substrate used in the caspase-3 assay could also be cleaved by caspase-7 [27], the activity measured in the caspase-3 assay will be referred to as caspase-3/7. One week after the beginning of RA treatment, activation of caspase-2, -3/7 and -9 was maximally increased by approximately 4, 7 and 3 fold, respectively. At day 14 the activity of each caspase had returned to the same level displayed by NT2 cells prior to RA treatment. Notably, not only a great fraction of caspase-2, -3/7 and -9 activity was associated with non-apoptotic (NA, Annexin V-negative) cells, but the time course of caspase activation was similar to that observed in the entire cell population (Figure 1, A-C). At day 7, the relative activity of caspase-2, -3/7 and -9 in non-apoptotic cells was +175%, +366% and +265%, respectively (P<0.05) of that measured in undifferentiated NT2 cells. On the other hand, the activity of caspase-1,-5,-6,-8 was not significantly affected throughout the differentiation process (Figure 1 D). These results indicate that caspase activation in differentiating NT2 cells is independent of apoptosis, that was found only in a small percentage of cells in the cultures (< 20%) and is known to occur during the first 3–4 days after RA induction [28]. Accordingly, no appreciable cleavage of poly-(ADP-ribose) polymerase (PARP), was detected at any time point during NT2 cell differentiation (Figure 2A). PARP is a known 116-kD substrate for caspase-3 and some caspase-3–like caspases that is cleaved during apoptosis to yield a 85 kDa fragment [29], [30].

Despite the similar time course, the relative activity of caspase-3/7 was twice that of caspase-2 and -9, suggesting that caspase-3 could be downstream of caspase-9 and/or -2 activation. To verify this hypothesis and elucidate the role of the two latter caspases in the RA-induced neuronal differentiation of NT2 cells, siRNA-mediated silencing of either caspase-9 (si-Casp9 cells) or caspase-2 (si-Casp2 cells) was carried out. Success of caspase-9 and caspase-2 silencing was confirmed by Western blotting analysis (Figure 2B and 2C, respectively), that showed a drastic reduction of each caspase expression at every time point examined.

### Caspase-9 Silencing Reduces the Expression of Neuronal Differentiation Markers

**Figure 3:**
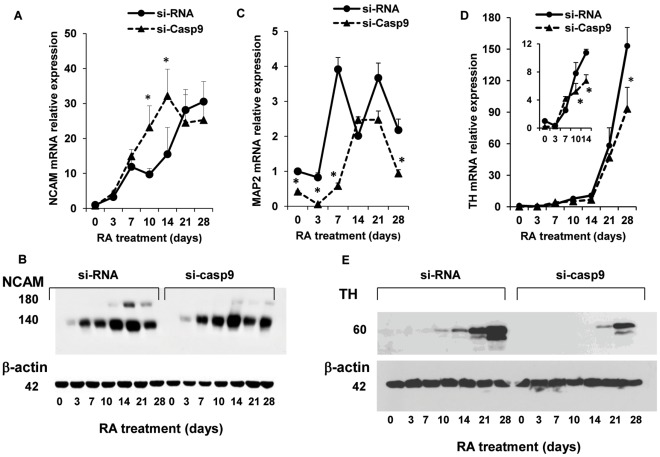
Caspase-9 silencing decreases the expression of neuronal markers in differentiating NT2 cells. NT2 cells stably transfected with scrambled si-RNA or with caspase-9 si-RNA (see Material and Methods section) were harvested at the indicated time points during RA treatment. A, C and D: relative expression of NCAM, MAP2 and TH mRNA, respectively, analyzed by real-time PCR. Results were normalized to GAPDH mRNA levels. For each neuronal marker, the expression in control undifferentiated NT2 cells (day 0) was set at a value of 1. Each point represents the mean ± SD of three experiments. B: representative western blots of NCAM protein expression in not interfered (si-RNA) and si-Casp-9 NT2 cells. The experiment was repeated three times with similar results. Densitometric analysis revealed, after normalization to actin, a decrease of NCAM-180 protein band to ∼10 and 40% of the intensity in control cells at day 21 and 28. E: representative western blots of TH protein expression in control (si-RNA) and si-Casp-9 cells showing that TH protein expression was both delayed and reduced in si-Casp9 cells, with respect to control cells. The doublets in the TH Western blots are likely the result of the expression of alternatively spliced TH mRNA. Inserts show on a smaller scale the results at the earlier time points of differentiation for MAP2 (insert 2C) and TH (insert 2D) mRNA expression. *P<0.05, versus the respective value in control NT2 cells.

With respect to control (scrambled si-RNA-transfected) NT2 cells, si-Casp9 cells displayed marked differences in the expression of neuronal differentiation markers (Figure 2). In control NT2 cells, RA induced a time dependent increase in the expression of NCAM mRNA, that by the third week reached a maximal level of expression of ∼25 fold (P<0.01), with respect to undifferentiated cells (Figure 3A). In si-Casp-9 cells the maximal expression of NCAM mRNA appeared earlier, day 10–14, and reached values higher than those in control cells, although NCAM mRNA expression was similar in control and si-Casp-9 cells at later time points (Figure 3A). The Western immunoblotting analysis of NCAM expression showed that, during RA-induced differentiation, NT2 cells expressed two major species of NCAM protein derived from mRNA alternative splicing [31]: NCAM-140 kDa that is detectable since day 3 from RA induction and NCAM-180 kDa that, in agreement with its higher expression in more differentiated neurons [32], begins to appear from day 14 and increases until day 21 of RA treatment (Figure 3B). While the NCAM-140 protein band was more intense than in control cells at day 7, 10 and 14 of RA treatment, the signal relative to NCAM-180 was markedly down-regulated in si-Casp-9 cells (Figure 3B). Silencing of caspase-9 also altered the level of MAP2 mRNA. In si-Casp-9 cells the expression of MAP2 mRNA was significantly reduced during the first week of treatment, when compared to control NT2 cells (Figure 3C). Although MAP2 mRNA expression was similar in si-Casp-9 and control cells at day 14, it was again lower at day 21 and 28 with respect to control cells (Figure 3C). NT2 cells differentiate into neurons endowed with different neurotransmitter phenotypes, including the dopaminergic one [33]. In control NT2 cells the mRNA of TH, the rate-limiting enzyme in dopamine synthesis, was induced by RA (∼2–3 fold) beginning at day 7 (Figure 3D). TH expression was ∼60 and 150 fold greater than in undifferentiated cells at day 21 and 28 of RA treatment, respectively. The mRNA expression of TH showed a similar time course in si-Casp-9 cells, but from day 7 onward was constantly lower than in control NT2 cells. TH mRNA reached a maximal increase of ∼90 fold at day 28, versus the respective undifferentiated cells, thus showing a ∼40% reduction (p<0.05) in comparison to control cells (Figure 3D). The TH protein expression in control NT2 cells appeared at day 10 of RA treatment and increased thereafter to reach its maximal expression at day 28. In si-Casp-9 cells the TH protein became visible only after 3 weeks of RA treatment and its expression was markedly lower than in control cells at day 28 (Figure 3E).

### Caspase-2 Silencing Increases the Expression of Neuronal Differentiation Markers

**Figure 4:**
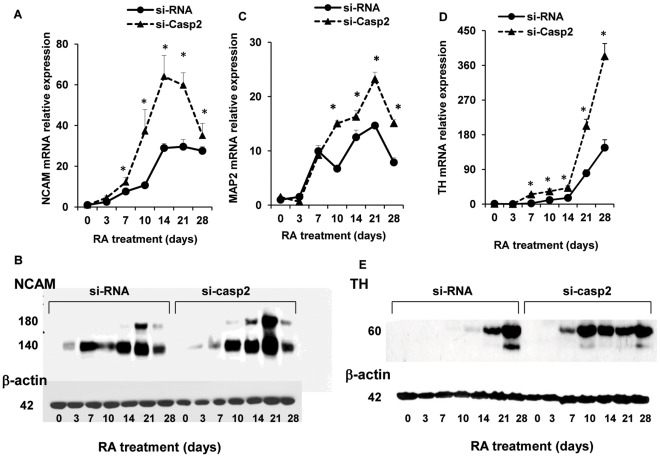
si-RNA-mediated caspase-2 silencing increases the expression of neuronal differentiation markers in differentiating NT2 cells. NT2 cells stably transfected with scrambled si-RNA or with caspase-2 si-RNA (see Material and Methods section) were harvested at the indicated time points during RA treatment. A, C and D: relative expression of NCAM, MAP2 and TH mRNA, respectively, analyzed by real-time PCR. Results were normalized to GAPDH mRNA levels. For each neuronal marker, the expression in control undifferentiated NT2 cells (day 0) was set at a value of 1. Each point represents the mean ± SD of three experiments. B: representative western blot of NCAM protein expression. Each blot was repeated three times with similar results. Densitometric analysis revealed, after normalization to actin, a ∼3, 32, 2.4 and 1.9 fold increase in the intensity of the NCAM-180 protein band in si-casp-2 cells, with respect to control cells at day 10, 14, 21 and 28, respectively. E: representative western blot of TH protein expression in control (si-RNA) and si-Casp-2 cells. The TH protein band appeared much earlier (7 versus 14 days) in si-Casp2 cells and densitometric analysis (n = 3) revealed higher intensity than in control cells. *P<0.05, versus the respective value control NT2 cells.

In si-Casp-2 cells the NCAM mRNA expression was higher (p<0.05) than in control cells at every time point, beginning at day 7 (Figure 4A). The higher expression of NCAM transcript in si-Casp2 cells was confirmed at the protein level: NCAM-140 was more abundantly expressed, particularly at day 10, 14 and 21, and NCAM-180 was markedly up-regulated at day 14, 21 and 28, respectively (Figure 4B). The apparent discrepancy between the NCAM mRNA, showing a peak at day 14, and the NCAM-180 protein level that appears to reach a peak in si-Casp2 cells at day 21, likely depends on the fact that the mRNA data include both NCAM-140 and NCAM-180 proteins, as the probe utilized in the real time PCR does not discriminate between the alternatively spliced mRNAs. The time course of MAP2 mRNA expression in si-Casp-2 cells was similar to that shown by control NT2 cells. Although showing a decreased expression at day 3 (−60%), MAP2 mRNA was up-regulated starting from day 10 of RA induction and was higher (P<0.05) than in control cells thereafter (Figure 4C). Also the TH mRNA expression was significantly up-regulated in si-Casp-2 cells starting at day 7 of RA treatment, when the difference was about 10 fold, and showed roughly twice the expression exhibited by control cells at the following time points (Figure 4D). Western blot analysis confirmed the earlier and higher expression of TH protein in si-Casp-2 cells, starting at day 7 and persisting up to day 28 (Figure 4E). Interestingly, the mRNA expression of the homeobox transcription factor Pitx-3, known to be expressed in a subset of dopaminergic neurons and to promote their maturation/survival [34], was decreased in si-Casp-9 cells but was 4 fold higher than in control cells at day 28 of RA treatment in si-Casp-2 cells (results not shown). As silencing of caspase-2 may have induced adaptive regulation of other caspases and particularly of caspase-9, we measured caspase-9 activity in si-Casp2 cells and found that was unchanged, with respect to control cells (data not shown).

### Silencing of Caspase-9, but not Caspase-2 Reduces Sirt1 Protein Cleavage and, Transiently, MASH1 Expression

**Figure 5:**
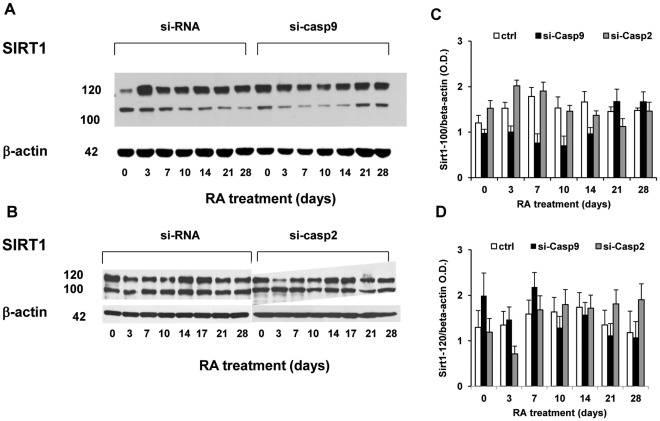
si-RNA-mediated silencing of caspase-9, but not of caspase-2, reduces Sirt1 cleavage. NT2 cells stably transfected with scrambled si-RNA, caspase-9 or caspase-2 si-RNA (see Material and Methods section) were harvested at the indicated time points during RA treatment. A: representative western blot of Sirt1 showing the ∼100 kDa fragment recognized by the anti-Sirt1 antibody (directed against an epitope in the C-terminus of the protein) in control and si-Casp9 cells during RA-induced differentiation. In si-Casp9 cells the intensity of the band corresponding to the 100 kDa Sirt1 fragment is clearly reduced between day 3 and 14 days. B: representative Sirt1 western blot in control and si-Casp2 cells, showing that the abundance of the 100 kDa Sirt1 fragment in si-Casp2 cells is comparable to that in control cells. C: results of densitometric analysis for the 100 kDa Sirt1 fragment (each bar represents the mean ± SD of 3 experiments). D: results of densitometric analysis for the full length Sirt1 (120 kDa, each bar represents the mean ± SD of 3 experiments), showing its constant expression in control, si-Casp9 and si-Casp2 cells.

**Figure 6:**
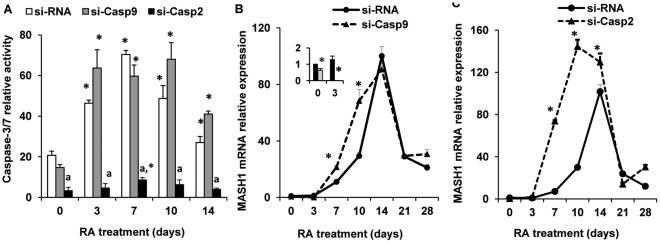
Effects of si-RNA-mediated silencing of caspase-9 and caspase-2 on Caspase-3/7 activity and MASH1 expression . A: caspase-3/7 activity assay showing that caspase-3/7 activation is similar in si-Casp9 and in control cells, while is markedly reduced in si-Casp2 cells. *P<0.05, versus the value in the respective undifferentiated cells (day 0); ^a^P<0.05, versus the respective value in control cells. B: relative expression of MASH1 analyzed by real-time PCR in control and si-Casp9 cells. Results were normalized to GAPDH mRNA levels. The MASH1 expression in control undifferentiated NT2 cells (day 0) was set at a value of 1. Each point represents the mean ± SD of three experiments. The insert shows on a smaller scale the results at day 0 and 3 of RA-induction. C: relative expression of MASH1 analyzed by real-time PCR in control and si-Casp2 cells. *P<0.05, versus the respective value in control NT2 cells.

Sirt1 is a NAD^+^-dependent class III histone deacetylase whose redox-dependent function controls neural precursor cell differentiation towards a glial or neuronal fate [19]. Because Sirt1 is also a putative substrate of several caspases in apoptotic conditions [20], we evaluated its expression during NT2 cell differentiation. The expression of the 120 kDa full length Sirt1 protein appeared rather constant during RA-induced differentiation (Figure 5A, B). The Western immunoblotting, however, showed that in addition to the full length protein, the anti-Sirt1 antibody (directed against a C-terminal epitope of Sirt1) identified a second immune-reactive band, migrating with an apparent m.w. of ∼100 kDa (Figure 5A,C). While in control cells the expression of the latter band showed a tendency to increase, between day 3 and 10, in si-Casp-9 cells was reduced between day 3 and 14 of RA treatment (Figure 5D), thus decreasing the ratio between the ∼100 kDa fragment and the full length Sirt1, that failed to significantly differ in comparison to control cells (Figure 5B). In contrast, the expression of the ∼100 kDa Sirt1 fragment was similar in si-Casp-2 cells and in control cells (Figure 5 C, D). As Sirt1 could be a substrate also for caspase-3 [20], the activity of the latter was measured in si-Casp-9 and si-Casp-2 cells. Caspase-3 activity before and during RA treatment, rather unexpectedly [5], was unchanged by caspase-9 silencing, but was markedly reduced in si-Casp2 cells (Figure 6A), thus pointing to a major role of caspase-9 in Sirt1 cleavage in differentiating NT2 cells.

Because the anti-Sirt1 antibody recognizes the Sirt1 protein C-terminus, it was reasonable to assume that the ∼100 kDa Sirt1 fragment visualized by our antibody lacks the N-terminus of the protein, and may thus represent an enzymatically/transcriptionally defective molecule. In murine neural precursor cells Sirt1 is recruited by the repressor bHLH factor(s) HES1/5 to the promoter of the proneural gene MASH1 and contributes to its repression [19]. We therefore tested whether the changes in Sirt1 cleavage observed in si-Casp-9 cells correlated with an altered expression of MASH1. As previously shown [35], MASH1 mRNA expression increased upon RA treatment in control cells, reaching a peak of expression (∼ 90 fold over the value in undifferentiated cells) at day 14 and substantially decreasing thereafter (Figure 6B and C). In si-Casp-9 cells MASH1 mRNA showed a similar time course, but its expression was markedly down-regulated at day 3 (P<0.05) (Figure 6B, insert). MASH1 expression in si-Casp-9 cells although higher at day 7 and 10, was similar to that of the respective control cells at later time points (Figure 6B). In contrast, in si-Casp-2 cells Sirt1 cleavage was effective (Figure 5C,D). Not only in si-Casp2 cells the RA-induced rise of MASH1 mRNA expression occurred 1 week earlier than in control cells, but up to day 14 its expression was markedly higher with respect to control NT2 cells (Figure 6C).

## Discussion

The present results show that caspase-2,-3 and -9 are selectively and transiently activated, in an apoptosis-independent manner, during the RA-induced neuronal differentiation of NT2 cells. Silencing of either caspase-9 or -2 shows that these two enzymes take part in the regulation of neuronal differentiation in an opposite manner: while caspase-9 activation facilitates, caspase-2 activation appears to hinder/delay the differentiation process, as shown by the downregulated or increased/earlier expression of neuronal differentiation markers in si-Casp9 or si-Casp-2 cells, respectively. The NAD^+^-dependent histone deacetylase Sirt1 is cleaved during NT2 neuronal differentiation in a caspase-9 dependent manner and the decrease of Sirt1 cleavage in si-Casp9 cells is associated with a transiently reduced expression of the proneural transcription factor MASH1 at early times of differentiation. Though, with respect to control cells, Sirt1 cleavage is not significantly affected in si-Casp2, the latter show an earlier and increased expression of MASH1 which is consistent with the earlier/higher expression of neuronal differentiation markers.

The timing of caspase activation and Sirt1 cleavage does not match the changes in neuronal marker expression, which last much longer. Such a temporal discrepancy suggests that the action of activated caspases on the expression of the neuronal differentiation markers examined, none of which to our knowledge been shown to behave as a caspase substrate, is indirect.

The activation of caspase-9 has been shown to play a crucial role in cellular differentiation and neuronal maturation through the cleavage/inactivation of gene products, i.e. Nanog, that support the multipotential and self-renewal features of stem cells [15] or semaphorin-7A, whose optimal concentration is important for the proper organization of mouse olfactory sensory neuron axonal projections [16]. The present results suggest that caspase-9 activation modulates neuronal differentiation/maturation also through the cleavage of Sirt1. In fact, caspase-9 silencing reduces the intensity of the anti-Sirt1 immunoreactive 100 kDa fragment between the beginning and day 14 of RA treatment. Thus, the present results extend the relevance of caspase-mediated Sirt1 cleavage, previously shown to occur under apoptotic conditions [20], and suggest its implication also in neuronal differentiation. As caspase-9 silencing does not alter caspase-3/7 activation in our cultures during differentiation (Figure 6A), the residual Sirt1 cleavage observed in si-Casp-9 cells may be due to the proteolitic action of caspase-3 [20]. The anti-Sirt1 antibody utilized in these experiments was generated against an epitope residing in the Sirt1 C-terminus, suggesting that the anti-Sirt1 immunoreactive fragment reasonably represents Sirt1 lacking the N-terminal region. The latter includes several putative caspase cleavage sites, i.e. ^121^DEDD^125^, ^163^DEED^166^ and ^214^ELDD^217^, that, however, need to be validated as physiological cleavage sites. Recently, Calvanese et al [18] showed that Sirt1 expression, measured by immunocytochemistry, is high in stem cells, where it correlates with the expression of pluripotency markers, and decreases upon differentiation. The fact that in our cultures full length Sirt1 and its fragment, assessed by western blot, failed to show reciprocal changes in their expression, though at first surprising, may reside in the fact that only a percentage of NT2 cells differentiate and not all of them are in the same differentiation stage at any given time point. Such an event would easily mask the actual protein changes occurring in distinct cell populations. Modeling by specific structural tests predicts long stretches of unordered regions at the N- and C-terminal portions of Sirt1, a feature that would confer flexibility and facilitate protein-protein interactions [36]. Indeed, a Sirt1 mutant lacking its N-terminal region fails to bind and deacetylate histone H1, with consequences on chromatin remodeling [37]. Alternatively, the N-terminus-lacking Sirt1 fragment detected in differentiating NT2 cells may represent a molecule unable to optimally interact with other proteins, either acting as Sirt1 regulators [17], [36] or endowed with DNA binding properties such as the bHLH transcription factor HES1 [38]. The lack of a stringent temporal correlation between Sirt1 cleavage and MASH1 expression and the rebound of the latter at day 10 and 14 of RA induction in si-Casp9 cells, cannot be easily explained at this moment. However, as the HES1/Sirt1 complex inhibits gene transcription of the neurogenic activator-type bHLH factor MASH1 [19], [38], the downregulation of MASH1 mRNA expression, observed in si-Casp9 cells at early times of differentiation, may be the consequence of a greater availability of full length Sirt1 for HES1 binding. Such an inference agrees with the recent demonstration that Sirt1 expression not only decreases upon differentiation of human and mouse embryonic stem cells, but also that the greatest changes were observed in neuroectodermal markers, that were overexpressed in Sirt1-knock-out cells and down-regulated in Sirt1 overexpressing cells [18]. The same authors, by showing a different time-course for the downregulation of Sirt1 mRNA, that decreases slowly, and Sirt1 protein, that drops markedly 7 days after differentiation induction, hypothesize that Sirt1 is regulated at various levels during human embryonic stem cell differentiation [18]. The present results suggest that non-apoptotic caspase-9-mediated cleavage is part of the regulatory network of Sirt1 functions. The fact that Sirt1 cleavage is present also in undifferentiated NT2 cells (Figure 5) may relate to the commitment of these cells towards the neuronal lineage [1]–[3]. Though previous evidence show that Sirt1 inhibition appears to regulate both positively [39] and negatively [40] neuronal differentiation, these discrepancies may be due to the different cell types examined or, alternatively, to the Sirt1 pleiotropic cellular functions, each likely requiring an appropriate dosage of its activity [17].

Notably, si-Casp-9 and si-Casp-2 cells show opposite changes in NCAM-180 protein expression which are paralleled by changes in the same direction in the expression of TH, a marker of the dopaminergic neuronal phenotype, and MAP2, a family of microtubule associated proteins (including MAP2 a and b) typically localized in the somato-dendritic compartment and important for conferring morphology and polarity to differentiated neurons [41]. A single NCAM gene is translated into three membrane-bound protein isoforms, NCAM-120, NCAM-140 and NCAM-180 kDa, by mRNA alternative splicing [31]. NCAM-120 is predominantly expressed in glia and, accordingly, was not observed in our cultures. As confirmed by the present results, NCAM-140 is most abundant during early neuronal differentiation, while NCAM-180 increases gradually during neuronal differentiation and is present in more differentiated/mature neurons [32], where its expression correlates with the establishment of stable synapses [42]. Accordingly, the expression of NCAM-180 was dramatically reduced in si-Casp9 cells, consistent with the reduced MAP2 and TH mRNA and protein expression, suggesting that caspase-9 silencing slowed down/decreased neuronal differentiation. In contrast, si-Casp2 cells exhibited both an early and enhanced expression of NCAM-180, in parallel with an increased expression of MAP2 and TH. Albeit through homophilic and heterophilic interactions NCAMs may promote neuronal differentiation and plasticity [31], a relationship between the changes of NCAM protein isoform expression and the caspase activation shown here does not appear straightforward as, to our knowledge, there is no indication that NCAMs are caspase substrates nor NCAM fragments were visualized in the western blots. However, histone acetylation-induced chromatin remodeling appears to cooperate with depolarization in the developmentally-regulated alternative splicing of NCAM mRNA [43]. It is therefore intriguing to speculate that the latter may be responsive to changes in Sirt1 deacetylase activity in si-Casp9 cells.

A puzzling aspect concerns the positive regulation exerted by si-RNA silencing of caspase-2 on NT2 neuronal differentiation, that hints to a detrimental physiological action of this caspase. Caspase-2 is the most evolutionarily conserved member of the family. Its high expression in neurons in the embryonic brain and decline during brain maturation led to infer that caspase-2 has a major role in neurodevelopment-associated apoptosis [44]. However, the precise role of caspase-2 in apoptosis and pathophyiology is, as yet, controversial due to the lack of identified specific substrates, the ambiguity of its function in the apoptotic cascade (initiator or executioner?) and the mild phenotype shown by caspase-2 null mice [45], [46]. The latter, in contrast to caspase-3 and -9 null mice characterized by embryonic or early post-natal death and brain abnormalities [47], develop normally and show limited and cell-type specific changes in sensitivity to apoptotic stimuli [48]. Remarkably, however, recent evidence shows that selective silencing/inhibition of caspase-2 exerts neuroprotective effects in adult and neonatal rodents exposed to ischemic insults [22]–[24]. Hence, as suggested [44], caspase-2 may perform cell-specific and context-dependent functions. For instance, caspase-2 is known to be activated in response to an increased production of reactive oxygen species/ROS [45], [46], a condition that also characterizes differentiating neurons from their progenitors [49]. Although the nature of caspase-2 putative substrate(s)/interacting partners in differentiating neurons is not known at the moment, the fact that caspase-2 is activated in non-apoptotic NT2 cells and that its silencing elicits an earlier and increased expression of neuronal differentiation markers suggests that caspase-2 activation is part of a homeostatic mechanism that regulate neuronal differentiation.

In conclusion, caspase-2 and -9 activation modulates in an opposite manner neuronal terminal differentiation/maturation, likely according to a complex and tightly-regulated program that appears to include, at least in part, Sirt1 functions. Although also caspase-3/7 activity was increased during RA-induced NT2 differentiation, such an activity was either unchanged, in si-Casp-9, or decreased, in si-Casp-2 cells. These results, while appearing at variance with previous reports on the relevance of caspase-3 for neuronal differentiation in murine neural precursors [12]–[16], also suggest that either caspase-3 has a minor role on the relatively “late” parameters of neuronal differentiation examined in our experimental model or that the activity we measured is mostly due to caspase-7. The latter, however, even though induced by RA at 48–72 hours in NT2 cells, would selectively degrade the proliferation-promoting factor OCT4 when NT2 cells were induced to differentiate by nucleoside drugs, but not by RA [50].

Altogether, although further experiments are needed to clarify the role of the increased caspase-3/7 activity, the present results extend the present knowledge regarding non-apoptotic functions of caspases in neuronal differentiation and point to distinct roles and time-frame of action for each caspase in the regulation of terminal neuronal differentiation. Such an issue appears relevant in the context of the potential use of caspase inhibitors [21]–[24] as a pharmacological approach to reduce the neuronal damage in neurodegenerative diseases that, on the other hand, may also benefit from stimulating or, at least, not counteracting the regenerative potential of resident neuronal progenitors.
